# Case report of renal manifestations in X-linked agammaglobulinemia

**DOI:** 10.3389/fimmu.2024.1376258

**Published:** 2024-07-25

**Authors:** Shuisen Wan, Meiling Cao, Jiahui Zou, Yaojia Bai, Mingyue Shi, Hongkun Jiang

**Affiliations:** ^1^ Department of Pediatrics, The First Hospital of China Medical University, Shenyang, Liaoning, China; ^2^ Department of Neonatology, The First Hospital of China Medical University, Shenyang, Liaoning, China

**Keywords:** inborn errors of immunity, primary antibody deficiency, renal disease, tubulointerstitial lesions, X-linked Agammaglobulinemia

## Abstract

**Introduction:**

X-linked agammaglobulinemia (XLA) is a humoral immunodeficiency disorder characterized by recurrent infections, severe hypogammaglobulinemia, and a deficiency of circulating B cells. While the hallmark clinical manifestations of XLA typically include the respiratory, dermatological, and gastrointestinal systems, renal involvement is infrequent. In this article, we report two cases of XLA with concurrent renal disease, supplemented with a review of documented cases.

**Case description:**

The two cases described involve twin brothers, both presenting with respiratory tract infections and renal manifestations. Subsequent genetic testing confirmed the diagnosis of XLA. The younger brother exhibited improvement following intravenous immunoglobulin (IVIG) therapy and anti-infection treatment. Due to financial constraints, the older brother received only anti-infection and symptomatic treatments. Seven months after discharge, the older brother developed nephritis. However, he showed improvement following IVIG treatment.

**Conclusion:**

Immune profiling and genetic testing should be considered in male children with recurrent infections to facilitate the effective diagnosis of XLA. Regular monitoring is also imperative to detect and treat immune-mediated renal diseases in patients with XLA.

## 1 Introduction

Inborn errors of immunity (IEI), previously referred to as primary immunodeficiency diseases (PID), constitute a spectrum of continuously evolving disorders primarily caused by single gene variants that induce alterations in the components and functions of immune cells and molecules. Clinical manifestations include heightened susceptibility to infections, autoimmune diseases, inflammatory conditions, allergies, bone marrow failure, and/or malignancies (1).

X-linked agammaglobulinemia (XLA) is a common primary antibody deficiency (PAD) within the spectrum of IEI, frequently diagnosed in pediatric practice (2). XLA is characterized by the most prevalent genetic anomaly resulting in impaired B cell development due to variants in the Bruton’s tyrosine kinase (*BTK*) gene, with an incidence estimated between 1:100,000 and 1:200,000 (3).

Key distinguishing features of XLA include increased vulnerability to infections accompanied by severe hypogammaglobulinemia and a deficiency of circulating peripheral blood B cells (B-cell percentage, < 2%). The *BTK* gene, located on the long arm of the X chromosome (Xq21.3–Xq22), spans a length of 37.5 kb and encodes a protein consisting of 659 amino acids. Comprising 19 exons, the *BTK* gene is susceptible to pathogenic variants in all structural domains and noncoding regions. The most common types include missense variants, followed by frame-shift variants, splice site variants, and nonsense variants, among others.

BTK, encoded by the *BTK* gene, belongs to the Tec family of non-receptor tyrosine kinases. BTK deficiency hinders the maturation of pro-B cells into later stages of pre-B cells in the B-cell differentiation pathway (4). Variants in the *BTK* gene disrupt B-cell development, leading to significantly reduced levels of mature B lymphocytes circulating in the peripheral blood of affected individuals. Consequently, this results in plasma cell generation disorders and a significant decrease across all subsets of B lymphocytes.

Approximately one-third of the cases of XLA demonstrate familial inheritance, while it is believed that new variants account for the remaining two-thirds (5). Although this condition predominantly affects men, women with extremely skewed X chromosome inactivation may exhibit similar clinical manifestations (6). Clinical onset is typically in early childhood, often after six months of age, coinciding with the weakening of maternal antibodies and the inability of affected individuals to produce their own immunoglobulins (7).

Patients with XLA may present with a diverse array of infections, encompassing both upper and lower respiratory tract infections, gastrointestinal infections, as well as invasive infections such as sepsis, meningitis, and osteomyelitis (7–12). While bacterial pathogens are primarily involved, viral and parasitic infections, including enteroviruses and *Giardia lamblia*, may also manifest (7–12).

Individuals diagnosed with XLA exhibit an elevated susceptibility to developing autoimmune diseases, with up to 15% of individuals experiencing conditions such as inflammatory bowel disease (IBD), autoimmune hemolytic anemia (AIHA), scleroderma, and immune complex-mediated glomerulonephritis (13–16). Additionally, reported cases document the co-occurrence of XLA with tumors (17, 18) and allergic rhinitis (19).

Once the diagnosis is established, immunoglobulin replacement therapy proves efficacious in managing XLA by significantly reducing infection rates and infection-associated complications, thereby improving patient survival rates (7–12).

Patients with XLA have deficiencies in humoral immunity, predisposing them to repeated infections, which in turn may lead to glomerular disease or tubulointerstitial disease in the context of infection and compromised immunity. However, detecting renal manifestations in patients with XLA can sometimes pose challenges as most relevant literature consists of case reports, and this makes it difficult to determine whether these renal findings represent sporadic occurrences or are linked to underlying pathophysiological mechanisms.

In this report, we have presented the clinical details of XLA in twin brothers presenting with renal manifestations, one of whom displayed a tubulointerstitial lesion on renal biopsy. Informed consent for the study was diligently obtained from the patients and their respective family members. Furthermore, we have incorporated a comprehensive review of the existing literature on renal biopsies conducted in patients with XLA to furnish a more detailed description of the clinical and pathological features as well as the prognostic implications associated with concurrent renal involvement.

## 2 Case descriptions

### 2.1 Case report 1

At the age of 11, the patient presented at another healthcare facility with symptoms of fever, coughing, and headaches. During his hospitalization, he developed gross hematuria, persisting for 16 days, accompanied by a red skin rash on both lower limbs. In order to establish a definitive diagnosis and receive further treatment, the patient was transferred to our medical center.

At the time of admission to our facility, multiple scattered nonpruritic red rashes that did not blanch upon pressure were observed on both lower limbs. Laboratory investigations revealed the following findings: urine analysis revealed that the urine-specific gravity was within the normal range, suggestive of glomerular origin (80% abnormal morphology of red blood cells) and the presence of microscopic hematuria (red blood cell 4/4/HP) as well as significant proteinuria (+++, 1.702 g/24 h). All five parameters for urinary microalbumin were elevated compared to normal levels: β2-microglobulin (β2-MG) was 3.47 mg/L (0–0.23 mg/L), α1-microglobulin (α1-MG) was 58.9 mg/L (0–12 mg/L), microalbuminuria (MA) was 289 mg/L (0–30 mg/L), urinary IgG was 12.3 mg/L (0–9.6 mg/L), and transferrin receptor protein TRU was 13.7 mg/L (0–2.41 mg/L). Peripheral blood test results showed that the white blood cell (WBC) level was 12.16*10^9^/L (4–10*10^9^/L), neutrophil (NE) was 8.19*10^9^/L (1.8–6.3*10^9^/L), hemoglobin (Hb) was 116 g/L (120–140 g/L), platelet (PLT) was 369*10^9^/L (100–300*10^9^/L), serum albumin (ALB) was 38.9 g/L (40–55 g/L), the urea concentration was 8.79 mmol/L (2.85–7.14 mmol/L), creatinine (Cr) was 114 µmol/L (59–104 µmol/L), cystatin C (Cys-C) was 2.28 mg/L (0.53–0.95 mg/L), estimated glomerular filtration rate (eGFR) was 53.80 mL/min/1.73 m^2^, lactate dehydrogenase (LDH) was 238 U/L (135–225 U/L), C-reactive protein (CRP) at 8.10 mg/L (0–5 mg/L), and procalcitonin (PCT) at 0.09 ng/mL (0–0.05 ng/mL).

Results of the immunological examination revealed that the patient had significantly decreased levels of immunoglobulins (IgG 2.08 g/L, IgA < 0.07 g/L, IgM < 0.04 g/L), a deficiency in B cells (CD19 percentage: 0%, CD19 absolute value: 0/µL), a reduced NK cell count (CD16 + 56 percentage: 3% [5%–27%], CD16 + 56 absolute value: 30/mL [90–590/mL]), and a decrease in the proportion of CD4^+^ T cells among T cells (CD4/CD8 ratio: 0.80). Other immunological parameters showed no significant abnormalities.

The blood lipid analysis and coagulation profile yielded normal results. A chest CT scan indicated localized bronchial dilation with mild chronic inflammation in the lower lobe of the left lung. A renal ultrasound revealed normal morphology with enhanced cortical echoes. Renal emission computed tomography (ECT) indicated normal blood perfusion but reduced renal parenchymal function and delayed excretion.

**Figure 1:**
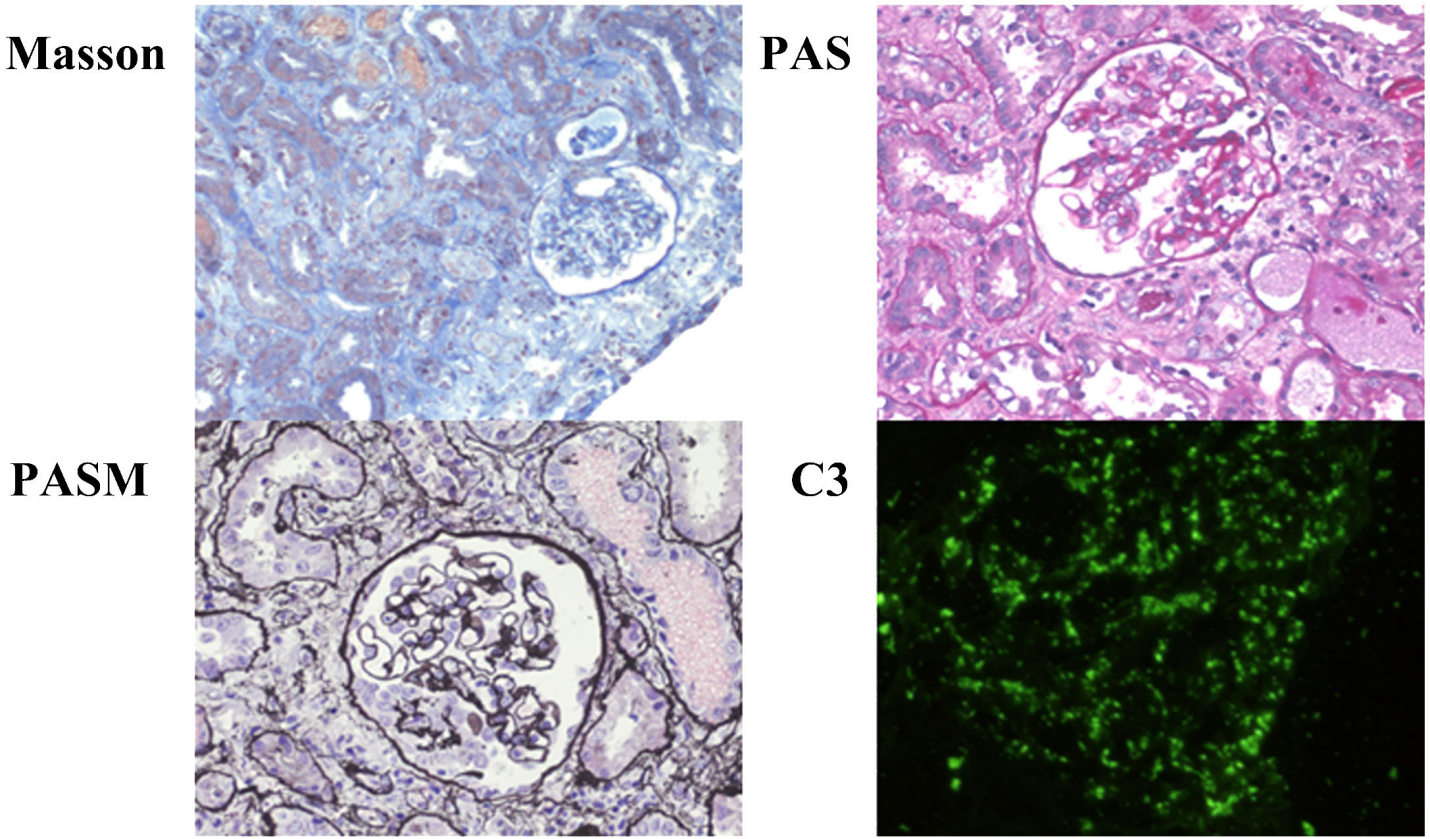
Renal biopsy of patient 1. Microscopic examination of renal biopsy reveals mild proliferation of mesangial cells and mesangial matrix in 16 glomeruli without obvious intraluminal proliferation. Occasional adhesions between glomerular capsules are observed, along with the formation of one-cell fibrous crescents showing focal necrosis. Extensive granular and vacuolar degeneration is observed in renal tubular epithelial cells, accompanied by detachment and exposure of basal membranes. Multi-focal tubular atrophy and luminal narrowing are present. Red blood cell casts frequently appear within the tubules. Interstitial edema is evident, along with the infiltration of numerous mononuclear cells in the intertubular spaces, accompanied by mild fibrosis. Immunofluorescence staining: C3 (+++) deposits appear arranged in a starry sky pattern within the glomeruli, IgA (−), IgM (−), Fib (−), IgG (−), and C1q (−).

Following admission, the child experienced recurrent fever with a maximum temperature of 39.2°C. Meropenem, combined with azithromycin, was intravenously administered for 3 days, after which the symptoms improved significantly. Renal biopsy findings were as follows (**Figure 1**): light microscopy revealed mild proliferation of mesangial cells and the mesangial matrix, with no obvious intraluminal proliferation. Occasional adhesion of glomerular tufts was observed, along with the presence of one fibrocellular crescent formation showing focal necrosis. Extensive granular and vacuolar degeneration was seen in the renal tubular epithelial cells, accompanied by desquamation and bare basement membrane formation. Multifocal atrophy and luminal narrowing of renal tubules were evident. Red blood cell casts were frequently observed within the tubular lumen. Interstitial edema, infiltration of individual nuclear cells in greater numbers between tubules, and mild fibrosis were also noted.

**Figure 2:**
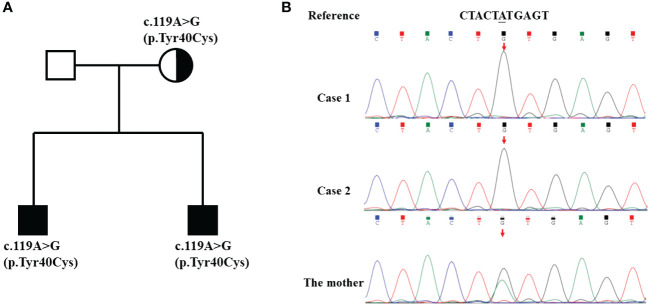
Pedigree and genomic sequence analysis of the *BTK* gene. **(A)** The pedigree of the family with XLA. The parents of the twins have no signs of XLA. **(B)** The c.119A>G mutation was detected in the twins and their mother.

Given the patient’s male sex and history of recurrent respiratory tract infections, two complete immunoglobulin tests were performed. The IgG, IgA, and IgM levels were all below 2 standard deviations from the mean values for children of the same age group. CD19+ B count was 0/µL. PID was suspected due to these findings as well as genetic testing results conducted on the patient, his mother, and his brother, revealing a variant in the *BTK* gene (p.Tyr40Cys) (**Figure 2**).

Based on the patient’s medical history and clinical examination findings, the confirmed diagnosis was as follows: (1) X-linked agammaglobulinemia, (2) acute renal injury (pathological diagnosis: C3 starry sky deposition, tubulointerstitial lesion), (3) renal insufficiency (decompensated phase), (4) community-acquired pneumonia with bronchiectasis, and (5) ventricular dilation (sequelae of encephalitis).

Treatment involved the administration of a total intravenous immunoglobulin (IVIG) dosage of 15 g over three consecutive days. Subsequent urine tests revealed negative proteinuria, a red blood cell count of 2/4/HP with abnormal morphology in 70% of cells, and a white blood cell count of 2–8/HP. The patient was discharged from the hospital, and a monthly IVIG at a dosage of 10 g was initiated. During the course of the IVIG treatment, the patient intermittently experienced respiratory infections and exhibited mild proteinuria and microscopic hematuria, which improved following the administration of gamma globulin and anti-inflammatory therapy.

### 2.2 Case report 2

An 11-year-old male patient, the twin brother of case 1, presented with a history of recurrent respiratory infections occurring five to six times per year and consistently recovering after intravenous administration of antibiotics. He was admitted to our department with symptoms including a cough, a runny nose persisting for 3 days, and a fever lasting for 2 days.

Urine analysis revealed the presence of microscopic hematuria (26/HP) and proteinuria (2+). Peripheral blood tests showed elevated WBC at 15.51*10^9^/L, neutrophil (NE#) at 12.14*10^9^/L, lymphocyte (LY#) at 1.50*10^9^/L, HBG at 130 g/L, PLT at 245*10^9^/L, iron level (Fe) at 3.9 µmol/L, CRP at 82.60 mg/L, PCT at 0.11 ng/mL, and IgE at 5.87 IU/mL. Immunological examination revealed significantly decreased levels of immunoglobulins: IgG, 1.53 g/L; IgA, < 0.01 g/L; and IgM, 0.03 g/L. Liver function, renal function, and myocardial enzymes were normal. A lung CT scan indicated scattered inflammation in both lungs.

The patient was administered intravenous cefixime and azithromycin, as well as nebulized budesonide, salbutamol, and interferon therapy. Subsequent to the oral administration of fosinopril sodium, the fever subsided and the cough improved. A urine retest showed +1 proteinuria but no red blood cells under high magnification.

Genetic testing of the patient, his younger brother, and his mother confirmed a diagnosis of X-linked agammaglobulinemia. Based on the medical history and clinical laboratory test results, the following specific diagnoses were made: (1) XLA, (2) acute pneumonia, and (3) acute glomerulonephritis. However, due to financial constraints at that time, the child was unable to receive immunoglobulin treatment.

The child presented with foamy urine and sought further evaluation at another medical facility 7 months postdischarge. Subsequent investigations revealed proteinuria (1.365 g/24 h) and microscopic hematuria (34.05/HP). Renal function tests and the renal ultrasound scan showed no abnormalities. A renal biopsy indicated membranoproliferative glomerulonephritis (image unavailable), characterized by mesangial cell proliferation, increased matrix deposition, and a thickened basement membrane with a double contour appearance under light microscopy. Additionally, immunofluorescence staining revealed C3 and IgG deposits in the mesangium and capillary loops.

The child was treated with antibiotics administered intravenously or orally, along with fosinopril sodium and IVIG therapy, resulting in symptomatic improvement. Due to the family’s financial constraints, only 12.5 g of IVIG was administered during the child’s hospitalization. Upon reevaluation, a routine urine test showed reduced hematuria and proteinuria compared to pretreatment levels. After discharge from the hospital, a monthly IVIG at a dosage of 12.5 g was initiated for the child. He experienced respiratory tract infections one to two times a year.

## 3 Discussion

A comprehensive summary of reported cases of XLA with concurrent renal disease that underwent renal biopsy, including the two cases that we examined, totaling 14 cases, is presented in **Table 1** (20–30). This summary includes details such as the age at the time of diagnosis of XLA, age at onset of renal manifestations, initial clinical presentations of renal involvement, relevant laboratory tests, physical examinations, clinical diagnoses, treatments administered, and prognosis. A summary of histopathological findings from renal biopsies in these cases, including light microscopy observations, immunofluorescence study results, electron microscopy outcomes, as well as pathological diagnoses and genetic testing results, is presented in **Table 2**.

**Table 1:** Initial renal manifestations, relevant examinations, diagnosis, treatment, and prognosis of XLA patients with renal biopsy.

NO.	Age at XLA diagnosis	Age at renal biopsy	Initial renalManifestations	Laboratoryexamination	Other examination	Diagnosis	Therapeuticinterventions	Clinical outcomes	Study
Case1	11	11	Gross hematuria and proteinuria	Proteinuria 1.702g/24h, SCR, 114 µmol/L; IgG, 3.09g/L; IgA, <0.07g/L; IgM, 0.07g/L	The thorax CT: consistent with bronchiectasis	XLA, AKI (tubulointers-titial lesions), renal insufficiency, pneumonia, ventricular dilatation	IVIG, antibiotics (meropenem, azithromycin), calcium dobesilate, compound α-keto acid	Asymptomatic with normal renal function and no proteinuria	——
Case2	11	12	Macroscopic hematuria and proteinuria	Proteinuria, 1.365g/24h; hematuria, 34.05/HP	Renal ultrasound revealed no abnormalities	XLA, MPGN	IVIG, antibiotics (cephalosporin)	Asymptomatic with hematuria and no proteinuria	——
1	16	16	Proteinuria and macroscopic hematuria	Proteinuria, 4.33 g/24 h; hematuria, 66.7/mL; IgG, 4.0 g/L; IgA, 0.38 g/L; IgM, < 0.188 g/L; IgE, 252 IU/mL, SCR, 69 μmol/L	High pressure (160/100 mmHg)	XLA, MPGN in CKD1 stage	Losartan	SCR, 72.5µmol/L; proteinuria, 2.16g/24h	Jiao et al. (20)
2	NM	NM	NM	NM	NM	XLA, proliferative immune-complex GN	NM	NM	Caza et al. (21)
3	NM	NM	NM	NM	NM	XLA, proliferative immune-complex GN	NM	NM	Caza et al. (21)
4	3	12	Progressive nephromegaly	IgG, 0.77 g/L; IgM, 0.09 g/L; IgA, 0.03 g; GFR, 50 mL/min/1.73 m^2^	Ultrasound: focal lesions in the liver, spleen, and kidneys, which were enlarged. Samples of kidney, liver, spleen, urine, CSF, and sputum: AiV1 positive	XLA, chronic Aichi virus infection	Splenectomy (13 y.o.), IVIG, nitazoxanide, itraconazole, liposomal amphotericin B, tacrolimus	Progressive kidney failure	Bucciol et al. (22)
5	0.5	11	Serum creatinine gradually increased without clinical symptoms	BUN 32.9mg/dL, SCR 2.19 mg/dL, CCR 40.1/ml/min/1.73m^2^, serum IgG trough 216 mg/dL	NM	XLA, IVIG-triggered tubulointerstitial nephritis	IVIG, methylprednisolone,F(ab') (2) fragment IgG , hemodialysis	Developed renal and liver failure and died at the age of 18.	Takeguchi et al. (23)
6	0.25	19	Mild renal dysfunction, proteinuria and macroscopic hematuria	SCR 1.5mg/dL, BUN 30mg/dL, proteinuria 0.14g/24h, CCR 39.2 mL/min/1.73 m^2^, IgG 685 mg/dL	NM	XLA, tubulointerstitial nephritis	IVIG, steroid therapy	Improved urinary findings and renal function	Sugimoto et al. (24)
7	3	33	Lower limb edema, weight loss, anemia, progressive deterioration of renal function and nephrotic range proteinuria	SCR, 1.67 mg/dL; Hb, 9.8 g/dL; proteinuria, 9540 mg/24 h	The thorax CT: consistent with bronchiectasis. Abdominal CT: a small nodule in the right hepatic lobe compatible with angioma	XLA, renal amyloidosis and bronchiectasis	IVIG, pharmacological treatment (no details), dialysis	On the waiting list for renal transplantation	Gonzalo-Garijo et al. (25)
8	0	10	Macroscopic hematuria and nephrotic proteinuria	IgG, 1.85 g/L; SCR, 32.71 mmol/L; ACR, 632.8 mg/mmol; C3, 58.7 mg/dL	NM	XLA, MPGN	IVIG, SCIG, prednisolone, ramipril	Asymptomatic with normal renal function and no proteinuria and improved hematuria	Lavrador et al. (26)
9	6	6	Persistent haematuria and proteinuria	IgG, 823mg/dL; SCR, 0.4mg/dL; proteinuria, 1.4g/24h	NM	XLA, lupus-like nephritis (WHO Class III), sinusitis	Prednisolone, chlorambucil, IVIG, mycophenolate mofetil	Asymptomatic with normal renal function and no proteinuria	Lim et al. (27)
10	5	25	Microscopic hematuria and proteinuria	IgG, 806mg/dL; IgM, 8mg/dL; IgA, <8mg/dL; proteinuria, 149mg/24h; SCR, 1.1 mg/dL	NM	XLA, MGP (stage II-III)	5 different preparations of IVIG	Asymptomatic with normal renal function and minimal proteinuria	Endo et al. (28)
11	6	12	Progressive oliguria and anuria	SCR, 2.3mg/dL; urea, 23mg/dL; K+, 7.2mEq/L	CT: bilateral, predominantly central, bronchiectasis with collapse of the left lower lobe	XLA, thrombocytopenia, ARDS, pneumonia, and ARF	Mechanical ventilation treatment, peritoneal dialysis, continuous renal replacement therapy	Died of cardiac arrest at the age of 12	BAL et al. (29)
12	0.17	4	Microscopic hematuria, mild proteinuria	U-pro/Cr, 0.83; C3, 58.7 mg/dL; SCR, 0.4mg/dL	NM	XLA, MPGN	IVIG, MPT	Improved hematuria and proteinuria and normalized serum complement levels	Yoshino et al. (30)

ACR, albumin/urine creatinine ratio; ARDS, acute respiratory distress syndrome; ARF, acute kidney failure; AKI, acute kidney injury; BUN, blood urea nitrogen; CCR, creatinine clearance rate; CSF, cerebrospinal fluid; CKD, chronic kidney disease; GFR, glomerularfiltration rate; GN, glomerulonephritis; IVIG, intravenous immunoglobulin; MGP, membranous glomerulopathy; MPGN, membranoproliferative glomerulonephritis; MPT, methylprednisolone pulse therapy; NM, not mentioned; SCIG, subcutaneous immunoglobulin; SCR, serum creatinine; XLA, X-linked agammaglobulinemia.

**Table 2:** Pathological findings of renal biopsy and genetic testing results.

NO.	Light microscope	Immunofluorescence	Electronic speculum	Genetic testing	Pathology diagnosis	Study
Case1	Mesangial cells and mesangial matrix were slightly proliferated, and a fibrous crescent was formed in one cell. There was extensive granular and vacuolar degeneration of renal tubular epithelial cells, multiple focal degeneration and atrophy of renal tubules, and luminal stenosis. The renal interstitium was edematous, with more mononuclear cell infiltration between tubules and mild fibrosis.	Glomerular deposition of C3	NA	BTK (p.Tyr40Cys)	Renal tubulointerstitial lesions	——
Case2	Mesangial cells and stroma proliferated, and thickening of the GBM and mesangial interposition were noted.	Mesangial and vascular loop deposits of C3 and IgG	NA	BTK (p.Tyr40Cys)	MPGN	——
1	Global glomerulosclerosis: 7.7%; segmental sclerosis: 7.7%. Mesangial cells and matrix increased, endothelial cells proliferated in many loops, and the segments of the cyst wall thickened and stratified. There were multiple small focal edema of renal tubular epithelial cells, a small amount of mononuclear cells and plasma cell infiltration in the interstitium, and a small amount of foam cell distribution. Hyaline degeneration of the arteriolar segments.	Mesangial area and vascular loop: IgG2+, IgG1+, IgG2 trace, IgG3++, IgG4 trace, IgM2+, C3+, C1qchain+, κ light chain+, λ light chain+, IgA: negative. PLA2R: negative	Electron-dense deposits in the mesangial area, epithelial side, and a small amount of subendothelium.	*BTK* (p.R82C)	MPGN, immune complex-mediated GN	Jiao et al. (20)
2	GGS: 36%; IF/TA, 20%–30%; AS: mild; AH: none	Capillary loop: Trace IgA, IgG 1+, C3 2+, mesangial +	NM	NM	Proliferative immune-complex GN(mesangial and/or endocapillary proliferative)	Caza et al. (21)
3	GGS: 12%; IF/TA: none; AS: none; AH: none	Capillary loop: trace IgA, IgG 3+, trace C3, C1q 1–2+, mesangial+	NM	NM	Proliferative immune-complex GN	Caza et al. (21)
4	The dense interstitial lymphoid infiltrate present between the remaining tubulin and glomeruli.	NM	NM	BTK(p.R28C)	NM	Bucciol et al. (22)
5	The first biopsy: severe interstitial accumulation of infiltrated lymphocytes. The glomerulus has a circumferential fibrocellular crescent. The second biopsy: severe interstitial fibrosis with infiltrated lymphocytes.	The first biopsy: mesangial deposits of IgG and C3. The second biopsy: no mesangial deposits of IgG or C3.	NM	BTK(g.55253_59624dupTGGCA)	NM	Takeguchi et al. (23)
6	Mononuclear cell infiltration in the interstitium loss of tubular epithelial cells, cloudy degeneration, and irregularity of the basement membrane in some renal tubules. Some glomeruli showed cellular crescent formation and fibrosclerotic lesions.	Granular deposition of IgG and C3 in the tubular basement membrane	Electron-dense deposits in the tubular basement membrane	BTK(p.Gln412X)	Tubulointerstitial nephritis	Sugimoto et al. (24)
7	Diffuse deposits of the variable intensity of an amorphous material, eosinophilic and PAS positive in glomeruli, mesangium, capillary walls, interstitium, peritubular areas, arteries, arterioles, and veins, with positive Congo red staining and apple-green birefringence under polarized light.	Focal and glomerular mesangial IgG deposits	NM	NM	Renal amyloidosis	Gonzalo-Garijo et al. (25)
8	Glomeruli with membranoproliferative patterns, without significant interstitial inflammation or tubular atrophy.	Glomerular deposition of IgG (+++), C3 (++), C4 (+), C1q (++), and traces of IgM in the mesangium and subendothelial space	NM	BTK (p.R288Q)	MPGN type I	Lavrador et al. (26)
9	A mild increase in the glomerular cellularity	Strong staining of IgG, IgA, C3, IgG κ, and λ in the mesangium and GBM with equivocal patterns of IgM and C1q	Diffuse foot process effacement and electronic dense deposits over the subendothelial, subepithelial, and paramesangial areas.	BTK (p.P116L)	Lupus-like nephritis (WHO Class III)	Lim et al. (27)
10	Increased mesangial matrix with mild segmental mesangial hypercellularity. The capillary basement membranes showed mild focal thickening.	Granular deposits along the capillary loops: IgGλ and IgGκ	An affected glomerulus revealed occasional subepithelial electron dense deposits.	A 10.8-kb tandem duplication of exons 6-18	MGP (stage II-III )	Endo et al. (28)
11	Shedding of the lining epithelial cells in the proximal tubules with granular casts in the lumen; some tubules with refractile fractured casts with epithelial reaction; the casts were faintly PAS-positive. A single focus of distal tubules showed the presence of yeast and pseudohyphae of Candida. The glomeruli, blood vessels and interstitium were within normal limits.	NM	NM	NM	ATN	BAL et al. (29)
12	The diffuse and global proliferation of mesangial cells, thickening of the GBM and mesangial interposition were noted.	Mesangium and GBM: strong staining of IgG, C3c and C3d, weak staining of C1q, C4 and IgA within the mesangium and GBM, but IgM was negative.	Effacement of the foot process and abundant subendothelial deposits together with some paramesangial and subepithelial deposits.	BTK(612insA)	MPGN type III	Yoshino et al. (30)

AH, arteriolar hyalinosis; AS, arteriosclerosis; ATN, acute tubular necrosis; GBM, glomerular basement membrane; GGS, global glomerulosclerosis; GN, glomerulonephritis; IF/TA, interstitial fibrosis/tubular atrophy; MGP, membranous glomerulopathy; MPGN,membranoproliferative glomerulonephritis; NA, not available; NM, not mentioned; PAS, periodic acid-Schiff; PLA2R, phospholipase A2 receptor.

The review involved 14 patients, including boys and adult men. The median age at diagnosis was 3 years (ranging from 0 to 16 years), while the median age at renal biopsy was 12 years (ranging from 4 to 33 years) (with some ages estimated based on information provided in the article). The common initial renal manifestations among all these cases comprised abnormal results in the urine analysis, such as proteinuria (nine of 14 cases; 64.3%) and hematuria (eight of 14 cases; 57.1%), along with decreased renal function (six of 14 cases; 42.9%). Out of the 14 patients, 13 underwent a renal biopsy, while a postmortem examination was done on one patient.

The most frequently observed pathological types detected in the examination of renal tissue were membranoproliferative glomerulonephritis (MPGN) and tubulointerstitial lesions. Other findings included immune complex-mediated glomerulonephritis, renal amyloidosis, membranous nephropathy, acute tubular necrosis, and lupus nephritis. Among the 14 cases, glomerulosclerosis was identified in four patients (28.6%), while mild interstitial fibrosis was present in three cases (21.4%), and crescent formation was noted in three cases (21.4%).

An immunofluorescence examination of renal tissues from 12 patients revealed the presence of immunoglobulin deposition in the majority of cases, including IgG (91.7%), IgA (33.3%), IgM (25.0%), C3 (83.3%), C1q (41.7%), and C4 (16.7%). The detection of immune complexes, immunoglobulins, and complement components in immunofluorescence suggested the involvement of humoral immunity in the observed renal manifestations. However, it remains unclear whether the deposited immunoglobulins originated from therapy or endogenous sources.

As shown in **Table 1**, patients with XLA and renal disease had low levels of immunoglobulins in their peripheral blood, and most of them received long-term IVIG treatment. In the cases reported by Takeguchi et al. (23), the initial renal biopsy confirmed the deposition of immunoglobulins and complement in glomeruli, while the subsequent renal biopsy after F(ab′)2 fragment IgG replacement therapy showed that these deposits had disappeared. Therefore, it was proposed that the deposition of immunoglobulins in glomeruli could originate from exogenous IVIG. Conversely, Jiao et al. (20) reported a case of MPGN occurring in a patient with XLA without prior immunoglobulin treatment, and they speculated that XLA itself could lead to MPGN, possibly due to dysregulation of peripheral B lymphocyte function and the production of abnormal endogenous antibodies. In our study, both twin patients had identical genotypes and experienced renal involvement before receiving immunoglobulin therapy, suggesting a plausible correlation between XLA and renal disease.

The standard initial dose of IVIG treatment for patients with XLA is typically 400–600 mg/kg per month, administered intravenously every three to four weeks or subcutaneously on a weekly or biweekly basis. The goal is to maintain serum IgG levels above 500 mg/dL in order to prevent potentially life-threatening recurrent bacterial infections (31, 32). However, long-term use of IVIG in patients with XLA necessitates careful monitoring for associated side effects. Apart from transient adverse reactions such as flushing, headache, fever, fatigue, and drowsiness, rare side effects such as renal damage, thrombosis formation, arrhythmias, aseptic meningitis, hemolytic anemia, and transfusion-related acute lung injury (TRALI) may also occur (33).

Renal impairment following IVIG may arise from several mechanisms: immune complex deposition in glomeruli, permeability nephritis due to immune-mediated hemolysis causing acute tubular obstruction, and transient vascular ischemia resulting from decreased renal perfusion (34–36). A minority of individuals with immunoglobulin-associated renal dysfunction progress to chronic renal failure or death (37, 38). These adverse reactions are contingent on the type of immunoglobulin preparation used and individual variations.

The current body of literature indicates that subcutaneous immunoglobulin (SCIG) demonstrates a reduced incidence of side effects, higher IgG trough levels, and lower infection rates in comparison to IVIG (39, 40). Further investigation is warranted to elucidate the source of immunoglobulin deposition in renal tissue and the associated immune mechanisms in XLA. In the management of patients with XLA receiving long-term IVIG therapy, timely evaluation of risk factors, administration of premedication, consideration of transitioning from IVIG to SCIG to minimize adverse reactions, and offering timely supportive treatment are crucial for optimizing the prognosis.

## 4 Conclusion

In conclusion, the following key points emerge:

1. Screening recommendations: Male children with recurrent infections should undergo thorough screenings for serum immunoglobulin, lymphocyte subsets, and other immune function evaluations. Additionally, genetic testing should be considered if warranted.
2. Genotype–phenotype correlation: The association between genotype and phenotype of XLA is unclear, as they may be influenced by factors such as gene modification, epigenetics, environment, or other unidentified variables.
3. Association with renal disease: XLA may be associated with immune-mediated renal diseases that often present with subtle or hidden phenotypes. Delayed diagnosis and delayed initiation of replacement therapy can lead to significant damage to renal function, precipitating various complications. For example, continued deterioration of renal function can result in the inability of the kidneys to excrete water, resulting in hypertension, pulmonary edema, and heart failure, which can be potentially fatal. In addition, susceptibility to infections, including urinary tract infections, lung infections, severe infections, and even sepsis, is heightened in affected individuals. Furthermore, in cases of renal failure, patients are prone to electrolyte imbalances such as hyperkalemia, posing a risk of cardiac arrest and death. Comprehensive renal-related examinations are crucial in patients with XLA, with prompt investigation of the immunopathogenesis for targeted therapeutic interventions.
4. Coexistence of autoimmune disease: XLA can also coexist with autoimmune diseases, potentially stemming from dysregulation of immune homeostasis triggered by genetic, immunological, and microbial factors, among others. B cells play a pivotal role in establishing long-term protective immunity.

## Ethics statement

Written informed consent was obtained from the minor(s)' legal guardian for the publication of any potentially identifiable images or data included in this article.

## Author contributions

SW: Data curation, Formal analysis, Software, Writing – original draft. MC: Conceptualization, Formal analysis, Writing – original draft. JZ: Data curation, Software, Writing – review & editing. YB: Formal analysis, Software, Writing – review & editing. MS: Data curation, Software, Writing – review & editing. HJ: Conceptualization, Funding acquisition, Project administration, Writing – review & editing.
